# Association of Innovations in Radiotherapy and Systemic Treatments With Clinical Outcomes in Patients With Melanoma Brain Metastasis From 2007 to 2016

**DOI:** 10.1001/jamanetworkopen.2020.8204

**Published:** 2020-07-06

**Authors:** Harry C. Brastianos, Paul Nguyen, Arjun Sahgal, Elizabeth A. Eisenhauer, Tara Baetz, Timothy P. Hanna

**Affiliations:** 1 Department of Oncology, Queen’s University, Kingston, Ontario, Canada; 2 ICES, Queen’s University, Kingston, Ontario, Canada; 3 Department of Radiation Oncology, Sunnybrook Odette Cancer Center, University of Toronto, Toronto, Ontario, Canada; 4 Division of Cancer Care and Epidemiology, Cancer Research Institute, Queen’s University, Kingston, Ontario, Canada

## Abstract

**Importance:**

Treatments for melanoma brain metastasis changed between 2007 and 2016 with the advent of new radiotherapy techniques, targeted therapeutic agents, and immunotherapy. Changes in clinical outcomes over time have not been systematically investigated in large population-based studies.

**Objective:**

To investigate the association of innovations in radiotherapy techniques and systemic therapies with clinical outcomes among patients with melanoma brain metastasis.

**Design, Setting and Participants:**

This population-based cohort study included all patients who received radiotherapy and/or surgery for the treatment of melanoma brain metastasis in Ontario, Canada, between January 1, 2007, and June 30, 2016. Brain treatment patterns and clinical outcomes were described by period (2007-2009, 2010-2012, and 2013-2016). Provincial administrative records were used to obtain data on surgery, radiotherapy, and drugs. Follow-up data were censored on August 31, 2016. A Kaplan-Meier analysis and multivariable Cox regression analyses were performed. Data were analyzed between November 8, 2017 and May 13, 2020.

**Main Outcomes and Measures:**

Overall survival, whole-brain radiotherapy-free survival, and time to subsequent brain treatment.

**Results:**

A total of 1096 patients (mean [SD] age, 61.7 [14.0] years; 751 men [68.5%]) with melanoma brain metastasis received treatment in Ontario between January 1, 2007, and June 30, 2016. Of those, 326 patients received treatment from 2007 to 2009 (period 1), 310 patients received treatment from 2010 to 2012 (period 2), and 460 patients received treatment from 2013 to 2016 (period 3). Patient age, other sociodemographic characteristics, and disease factors were similar between periods. Whole-brain radiotherapy was the first local brain-directed treatment used in 246 patients (75.5%; 95% CI, 70.8%-80.1%) in period 1, decreasing to 239 patients (52.0%; 95% CI, 47.4%-56.5%) in period 3. The use of partial-brain radiotherapy techniques as the first treatment increased from 11 patients (3.4%; 95% CI, 1.4%-5.3%) in period 1 to 98 patients (21.3%; 95% CI, 17.6%-25.0%) in period 3. After the first treatment for melanoma brain metastasis, the use of BRAF and MEK inhibitors and immunotherapy increased from less than 6 patients (<1.8%; 95% CI, <0.4% to <3.3%) in period 1 to 188 patients (40.9%; 95% CI, 36.4%-45.4%) in period 3. Overall survival was greater in period 3 (1-year survival, 21.8% [95% CI, 17.9%-25.9%] and 2-year survival, 13.8% [95% CI, 10.4%-17.8%]; Wilcoxon *P* = .001) compared with period 1 (1-year survival, 12.3% [95% CI, 9.0%-16.1%] and 2-year survival, 6.4% [95% CI, 4.1%-9.5%]), with an adjusted hazard ratio (aHR) of 0.65 (95% CI, 0.56-0.77). The findings were unchanged after accounting for the variation in imaging practice between periods. Between period 1 and period 3, the use of whole-brain radiotherapy decreased (aHR, 0.32; 95% CI, 0.22-0.46), and the use of multiple brain-directed treatments increased (aHR, 2.16; 95% CI, 1.48-3.14).

**Conclusions and Relevance:**

These findings suggest that innovations in systemic therapy and radiotherapy are associated with improvements in clinical outcomes among patients with melanoma brain metastasis, even in population-wide routine practice. Overall survival improved over time, and the use of whole-brain radiotherapy decreased. However, many patients continued to receive whole-brain radiotherapy in the last period (2013-2016) examined.

## Key Points

**Question:**

Are innovations in the treatment of melanoma brain metastasis associated with improvements in clinical outcomes at the population level?

**Findings:**

In this cohort study of 1096 patients with melanoma brain metastasis between 2007 and 2016, the use of advanced radiotherapy techniques, targeted therapies, and immunotherapies increased over time. Treatment with whole-brain radiotherapy decreased and overall survival increased, and the association between the use of new techniques and therapies for the treatment of melanoma brain metastasis and improvements in clinical outcomes remained unchanged when the analyses were adjusted for patient, disease, and time factors.

**Meaning:**

The study’s findings suggest that innovations in the treatment of melanoma brain metastasis are associated with improvements in outcomes among the group of patients with melanoma who have the worst prognosis based on population-wide routine practice.

## Teaser

This cohort study examines the association of innovations in radiotherapy techniques and systemic treatment with clinical outcomes among Canadian patients with melanoma brain metastasis between 2007 and 2016.

## Introduction

The management of patients with melanoma brain metastasis has evolved over the last decade (eFigure 1 in the Supplement). As early as 2006, data from randomized clinical trials indicated that treatment with whole-brain radiotherapy (WBRT) combined with stereotactic radiosurgery (SRS) was not associated with improvements in survival compared with treatment with SRS alone.^1^ Additional studies were consistent with this finding, and they reported that the addition of WBRT to SRS was associated with a more substantial decrease in learning and memory function.^2,3,4^ The findings of these studies resulted in changes to the standard of care for patients with brain metastasis, with SRS used solely without WBRT^5^ to treat patients with a diagnosis of limited brain metastasis.

The guidelines for systemic therapy for the treatment of metastatic melanoma have also evolved. In 2009, the guidelines recommended that cytotoxic chemotherapies, such as dacarbazine, be offered to patients with metastatic melanoma.^6,7^ For patients with brain metastasis, temozolomide was an option.^8^ Starting in 2010, the landscape of melanoma treatment changed with the development of checkpoint inhibitors and targeted therapeutic agents.^9,10^ Moreover, many of these treatments were found to have activity in patients with brain metastasis.^11,12^

For patients with melanoma brain metastasis, improvements in clinical outcomes associated with innovations in both radiotherapy and systemic therapy have not been systematically investigated in large population-based studies that include all patients. We undertook a population-based study to investigate changes in the treatment management and clinical outcomes of all patients with melanoma who received treatment for brain metastasis in the Canadian province of Ontario between 2007 and 2016.

## Methods

### Study Population

This population-based study included all patients with melanoma who received local radiotherapy and/or surgery for brain metastasis in Ontario, Canada, between January 1, 2007, and June 30, 2016. The study cohort was divided into 3 periods based on the date of the first local brain-directed treatment: period 1 (2007-2009), period 2 (2010-2012), and period 3 (2013-2016). The study was approved by the Queen’s University Research Ethics Board with a waiver of informed consent based on the Personal Health Information Protection Act, Section 44(1), which allows waivers of informed consent for research meeting criteria including whether the research can be accomplished without the requested personal health information, research that is in the public interest, safeguards for privacy, and impracticality of obtaining informed consent. This study followed the Strengthening the Reporting of Observational Studies in Epidemiology (STROBE) reporting guideline for cohort studies.

Patients with a diagnosis of cutaneous melanoma were identified through the Ontario Cancer Registry. Patients with other cancer diagnoses in addition to melanoma were excluded to minimize the risk of misclassifying treatment information. Ontario has a population of 14.6 million and uses a single-payer universal health care system. Treatments delivered to patients, including intravenous systemic therapy, radiotherapy, and surgery, are coordinated through Cancer Care Ontario and provided through a single-payer publicly funded system. Treatment is regionalized and largely delivered through cancer centers and regional cancer programs distributed among local health integration networks. Oral medications are provided through various programs but administered to patients through the publicly funded cancer care system.

### Data Sources

The Ontario Cancer Registry is a population-based cancer registry that captures both demographic and diagnostic data from a large percentage of the population, including patients with melanoma.^13,14^ Data regarding surgical procedures were obtained from the Canadian Institute for Health Information hospital database. Information about radiotherapy was obtained from the records of all cancer centers. Physician billing data, provincial records of chemotherapy delivery, and treatment records from all regional cancer centers were used to identify chemotherapy use. All data sets were linked using unique encoded identifiers and were analyzed at ICES (previously the Institute for Clinical Evaluative Sciences).

The new systemic therapies were categorized as regimens that contained targeted therapeutic agents (eg, BRAF and MEK inhibitors), anti–PD-1 (anti–programmed cell death 1) monoclonal antibodies, anti–cytotoxic T-lymphocyte antigen 4 (anti–CTLA-4) antibodies, or other new drugs, including those used in clinical trials. Cytotoxic agents, such as dacarbazine and temozolomide, were separately classified. Several satellite centers that delivered chemotherapy may not have reported the drug treatments directly to Cancer Care Ontario. To ensure our data were as complete as possible, provincial reimbursement data from specialist physicians delivering therapy were used to identify systemic therapies not reported in the drug databases. These data included a specific code for oral systemic therapy that was introduced in 2011.

### Classification of Variables

We investigated outcomes according to the period during which patients received their first brain-directed treatment (2007-2009, 2010-2012, and 2013-2016). We described and adjusted for patient characteristics at the time of the first local brain-directed treatment, melanoma diagnosis characteristics, and time factors. Patient factors included age, sex, socioeconomic status, rurality of residence, and comorbidity. Socioeconomic status was based on community-specific or neighborhood household income quintiles generated by quintile rankings of the neighborhood average income per single person equivalent within each census metropolitan area or census agglomeration (quintile range, 1-5, with 1 indicating the lowest quintile and 5 indicating the highest quintile of income per person equivalent within each census metropolitan area or census agglomeration).^15^

Rurality of patient residence at the time of their first treatment for melanoma brain metastasis was characterized using the 2008 Rurality Index for Ontario scale (scale range, 0-100, with 0 indicating a low degree of rurality and 100 indicating a high degree of rurality).^16^ The Elixhauser Comorbidity Index score (score range, 0 to 31, with 0 indicating no identified comorbidity and one point added per additional comorbidity) was determined using data from the Canadian Institute for Health Information Discharge Abstract Database and the Same Day Surgery Database.^17^ Diagnostic codes for cancer metastasis or solid tumor without metastasis were not included in the comorbidity score. To ensure the data on comorbidity status were complete, patients who had lapses in their provincial health coverage 5 years before the first part of treatment were not included. Disease factors were melanoma morphology and body site. Time factors included time from the first diagnosis of any stage of melanoma to the first palliative treatment and time from the first palliative treatment to the first brain-directed treatment.

Overall survival was measured from the time of the first local brain-directed therapy. Secondary end points were WBRT-free survival and the time from 6 weeks after the first brain-directed therapy to subsequent brain treatment. Follow-up data were censored on August 31, 2016.

### Statistical Analysis

Kaplan-Meier methods were used to analyze time-to-event data. For the subgroup who received a first brain treatment (radiotherapy or surgery) that was limited to visible brain lesions (ie, non-WBRT treatment), WBRT-free survival was defined as the duration of survival without WBRT. Deaths associated with any cause were censored. Log rank and Gehan-Breslow-Wilcoxon tests were used, and a Cox proportional hazards regression model analysis was performed, with adjustments for patient, disease, and time factors. To evaluate interaction effects in the Cox model adjusted for imaging practice, joint tests with full-rank parameters were used. The significance threshold for all analyses was *P* < .05. Data analyses were performed between November 8, 2017, and May 13, 2020, using SAS software, version 9.4 (SAS Institute).

## Results

A total of 1096 patients with melanoma (mean [SD] age, 61.7 [14.0] years; 751 men [68.5%] and 345 women [31.5%]) received treatment with local brain-directed therapy that included either radiotherapy, surgical excision, or both between January 1, 2007, and June 30, 2016 (Table 1; eFigure 2 in the Supplement). Of those, 326 patients received treatment in period 1 (2007-2009), 310 patients received treatment in period 2 (2010-2012), and 460 patients received treatment in period 3 (2013-2016). The mean age of patients was similar between periods. More patients lived in neighborhoods in the upper-income quintiles (245 patients [22.4%; 95% CI, 19.9%-24.8%] in quintile 4 and 257 patients [23.5%; 95% CI, 20.9%-26.0%] in quintile 5) and in urban centers (684 patients [62.4%; 95% CI, 59.5%-65.3%]). The median time between the first melanoma diagnosis and the first palliative treatment (systemic therapy, radiotherapy, and/or metastasis surgery) was 23 months (interquartile range [IQR], 6-55 months), with little difference between periods. The median time between the first palliative treatment and the first brain-directed treatment was the same between periods (0 months), although the mean time was greater in periods 2 and 3 (mean [SD] time, 100.3 [216.6] days and 110.6 [225.5] days, respectively) compared with period 1 (mean [SD] time, 53.5 [131.9] days; *P* < .001). We observed an increase in the annual number of patients with brain metastases and an increase in the overall incidence of melanoma in Ontario^18^ over time (in 2007, 1909 melanoma incidence cases, and 96 with initial brain-directed treatment; in 2015, 2437 melanoma incidence cases, and 144 with initial brain-directed treatment).

**Table 1.** Patient Characteristics

Characteristic	No. (%)			
	Year of first brain treatment			Total
	2007-2009	2010-2012	2013-2016	
Total No.	326	310	460	1096
Age, y				
Mean (SD)	61.3 (14.4)	61.2 (14.2)	62.3 (13.6)	61.7 (14.0)
Median (IQR)	62 (50-73)	61 (51-73)	62 (54-72)	62 (52-73)
Category				
20-39	26 (8.0)	21 (6.8)	29 (6.3)	76 (6.9)
40-49	48 (14.7)	43 (13.9)	46 (10.0)	137 (12.5)
50-59	71 (21.8)	74 (23.9)	116 (25.2)	261 (23.8)
60-69	76 (23.3)	81 (26.1)	119 (25.9)	276 (25.2)
70-79	71 (21.8)	56 (18.1)	103 (22.4)	230 (21.0)
≥80	34 (10.4)	35 (11.3)	47 (10.2)	116 (10.6)
Sex				
Female	97 (29.8)	113 (36.5)	135 (29.4)	345 (31.5)
Male	229 (70.3)	197 (63.6)	325 (70.7)	751 (68.5)
Elixhauser Comorbidity Index score^a^				
0	175 (53.7)	177 (57.1)	244 (53.0)	596 (54.4)
1	79 (24.2)	75 (24.2)	108 (23.5)	262 (23.9)
2	35 (10.7)	34 (11.0)	50 (10.9)	119 (10.9)
≥3	37 (11.4)	24 (7.7)	58 (12.6)	119 (10.9)
Neighborhood income, quintile^b^				
1 or missing	59 (18.1)	51 (16.5)	76 (16.5)	186 (17.0)
2	66 (20.3)	55 (17.7)	81 (17.6)	202 (18.4)
3	59 (18.1)	52 (16.8)	95 (20.7)	206 (18.8)
4	72 (22.1)	71 (22.9)	102 (22.2)	245 (22.4)
5	70 (21.5)	81 (26.1)	106 (23.0)	257 (23.5)
Rurality Index for Ontario^c^				
Urban (<10)	213 (65.3)	206 (66.5)	265 (57.6)	684 (62.4)
Suburban (10 to <40)	80 (24.5)	85 (27.4)	142 (30.9)	307 (28.0)
Rural or missing (≥40)	33 (10.1)	19 (6.1)	53 (11.5)	105 (9.6)
Morphology of first melanoma diagnosis				
Nodular	54 (16.6)	73 (23.6)	130 (28.3)	257 (23.5)
Acral lentiginous	8 (2.5)	6 (1.9)	12 (2.6)	26 (2.4)
Desmoplastic or lentigo maligna	14 (4.3)	8 (2.6)	19 (4.1)	41 (3.7)
Superficial spreading	49 (15.0)	63 (20.3)	94 (20.4)	206 (18.8)
Not otherwise specified	182 (55.8)	136 (43.9)	186 (40.4)	504 (46.0)
Multiple primaries or other	19 (5.8)	24 (7.7)	19 (4.1)	62 (5.7)
Topography of first melanoma diagnosis				
External ear	6 (1.8)	8 (2.6)	16 (3.5)	30 (2.7)
Lower limb and hip	54 (16.6)	48 (15.5)	57 (12.4)	159 (14.5)
Scalp and neck	21 (6.4)	28 (9.0)	40 (8.7)	89 (8.1)
Trunk	103 (31.6)	106 (34.2)	166 (36.1)	375 (34.2)
Upper limb and shoulder	42 (12.9)	55 (17.7)	81 (17.6)	178 (16.2)
Other parts of face	21 (6.4)	16 (5.2)	27 (5.9)	64 (5.8)
Malignant neoplasm of skin (site unspecified) or multiple primaries	79 (24.2)	49 (15.8)	73 (15.9)	201 (18.3)
Time between first melanoma diagnosis and first palliative treatment, mo				
Mean (SD)	44.0 (63.8)	49.6 (67.2)	45.1 (63.2)	46.0 (64.5)
Median (IQR)	23 (5-51)	26 (9-58)	21 (6-58)	23 (6-55)
Category				
0-5	85 (26.1)	63 (20.3)	110 (23.9)	258 (23.5)
6-11	30 (9.2)	26 (8.4)	45 (9.8)	101 (9.2)
12-17	34 (10.4)	36 (11.6)	40 (8.7)	110 (10.0)
18-23	18 (5.5)	23 (7.4)	47 (10.2)	88 (8.0)
24-35	37 (11.4)	41 (13.2)	49 (10.7)	127 (11.6)
36-59	51 (15.6)	46 (14.8)	59 (12.8)	156 (14.2)
60-119	44 (13.5)	40 (12.9)	70 (15.2)	154 (14.1)
≥120	27 (8.3)	35 (11.3)	40 (8.7)	102 (9.3)
Time between first palliative treatment and first brain-directed treatment, d				
Mean (SD)	53.5 (131.9)	100.3 (216.6)	110.6 (225.5)	90.69 (200.8)
Median (IQR)	0 (0-15)	0 (0-104)	0 (0-135)	0 (0-91)
Category, mo				
At first palliative treatment	239 (73.3)	205 (66.1)	269 (58.5)	713 (65.1)
0-2	33 (10.1)	25 (8.1)	49 (10.7)	107 (9.8)
3-5	17 (5.2)	22 (7.1)	48 (10.4)	87 (7.9)
6-11	22 (6.8)	28 (9.0)	48 (10.4)	98 (8.9)
≥12	15 (4.6)	30 (9.7)	46 (10.0)	91 (8.3)

Abbreviation: IQR, interquartile range.

^a^ Score range, 0 to 31, with 0 indicating no identified comorbidity and one point added per additional comorbidity.

^b^ Quintile range, 1-5, with 1 indicating the lowest quintile and 5 indicating the highest quintile of the community-specific average income per single person equivalent within each census metropolitan area or census agglomeration. Quintile rankings were defined within each area to better reflect the relative nature of household income and minimize the consequences of large differences in housing costs for household welfare.

^c^ Scale range, 0-100, with 0 indicating a low degree of rurality and 100 indicating a high degree of rurality.

The type of systemic agent used for treatment changed over time (Table 2). From the time of the first palliative treatment to the time of last follow-up or death, a substantial increase in the use of new therapeutic agents was observed, with 8 patients (2.5%; 95% CI, 0.8%-4.1%) receiving BRAF and MEK inhibitor therapies or immunotherapies in period 1 compared with 247 patients (53.7%; 95% CI, 49.1%-58.3%) in period 3 (*P* < .001). After the first treatment for melanoma brain metastasis, the use of BRAF and MEK inhibitors and immunotherapy increased from less than 6 patients (<1.8%; 95% CI, <0.4% to <3.3%) in period 1 to 188 patients (40.9%; 95% CI, 36.4%-45.4%) in period 3 (*P* < .001).

**Table 2.** Systemic Treatment Delivered

Treatment^**a**^	No. (%)^**b**^			
	Year of first brain treatment			Total
	2007-2009	2010-2012	2013-2016	
Total No.	**326**	**310**	**460**	1096
Before first brain-directed therapy				
Adjuvant interferon	66 (20.3)	84 (27.1)	87 (18.9)	237 (21.6)
BRAF/MEK inhibitor	0	19 (6.1)	56 (12.2)	75 (6.8)
Immunotherapy (CTLA-4/PD-1)	<6 (<1.8)	25 (8.1)	81 (17.6)	<112 (<10.2)
Temozolomide	7 (2.2)	<6 (<1.9)	<6 (<1.3)	<19 (<1.7)
Other named systemic therapy^c^	47 (14.4)	70 (22.6)	65 (14.1)	182 (16.6)
BRAF/MEK inhibitor or immunotherapy	<6 (<1.8)	39 (12.6)	118 (25.7)	<163 (<14.9)
BRAF/MEK inhibitor, immunotherapy, or oral systemic agent reimbursement	<6 (<1.8)	42 (13.6)	137 (29.8)	<185 (<16.9)
From first brain-directed therapy until death or censoring				
BRAF/MEK inhibitor	<6 (<1.8)	20 (6.5)	73 (15.9)	<99 (<9.0)
Immunotherapy (CTLA-4/PD-1)	<6 (<1.8)	35 (11.3)	140 (30.4)	<181 (<16.5)
Temozolomide	35 (10.7)	28 (9.0)	12 (2.6)	75 (6.8)
Other named systemic therapy^c^	32 (9.8)	47 (15.2)	38 (8.3)	117 (10.7)
BRAF/MEK inhibitor or immunotherapy	<6 (<1.8)	51 (16.5)	188 (40.9)	<245 (<22.4)
BRAF/MEK inhibitor, immunotherapy, or oral systemic agent reimbursement	6 (1.8)	58 (18.7)	197 (42.8)	261 (23.8)
From first palliative treatment until death or censoring				
BRAF/MEK inhibitor	<6 (<1.8)	38 (12.3)	113 (24.6)	<157 (<14.3)
Immunotherapy (CTLA-4/PD-1)	8 (2.5)	56 (18.1)	182 (39.6)	246 (22.5)
Temozolomide	40 (12.3)	32 (10.3)	15 (3.3)	87 (7.9)
Other named systemic therapy^c^	72 (22.1)	104 (33.6)	97 (21.1)	273 (24.9)
BRAF/MEK inhibitor or immunotherapy	8 (2.5)	82 (26.5)	247 (53.7)	337 (30.8)
BRAF/MEK inhibitor, immunotherapy, or oral systemic agent reimbursement	10 (3.1)	92 (29.7)	268 (58.3)	370 (33.8)

Abbreviation: CTLA-4, cytotoxic T-lymphocyte antigen 4; PD-1, programmed cell death 1.

^a^ Some patients may have received more than 1 class of systemic therapy.

^b^ In accordance with administrative data privacy regulations, information was not reported for groups with 1 to 5 patients.

^c^ Other named systemic therapy include dacarbazine, carbo-taxol agents, and clinical trial regimens.

**Figure 1.**
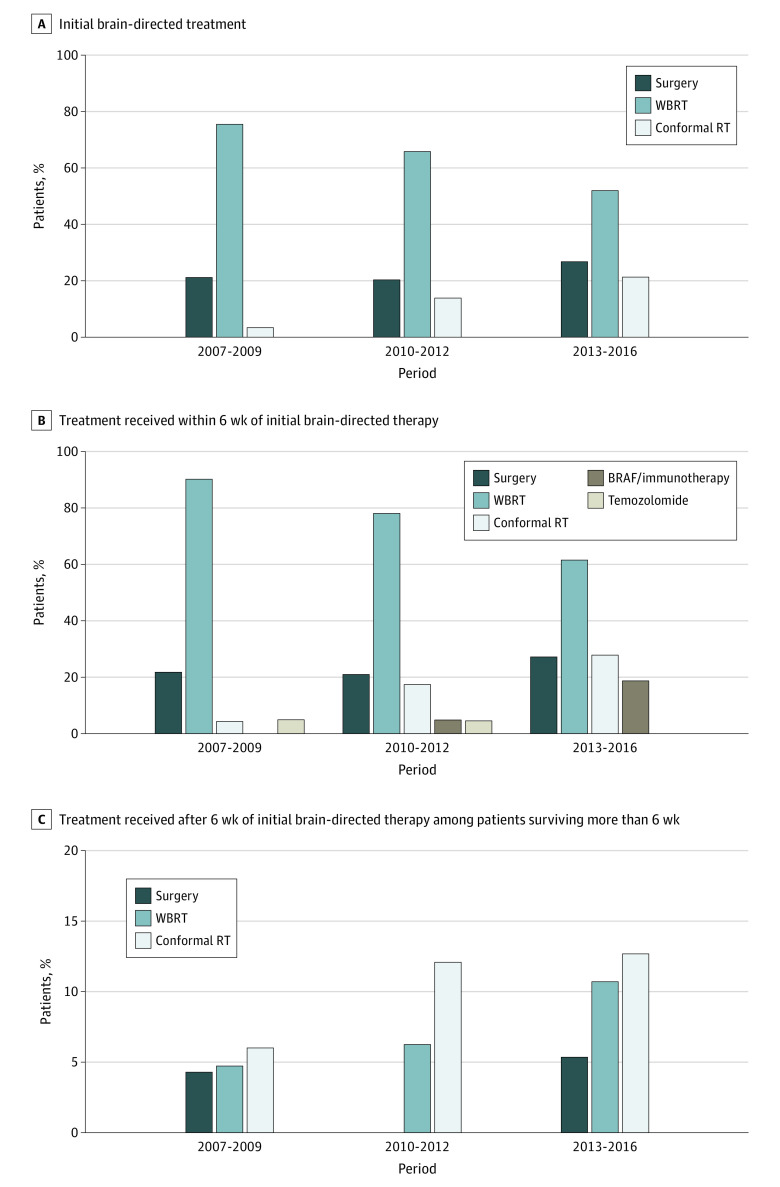
Temporal Trends in Melanoma Brain Metastasis Treatment Axes reflect the data range and are therefore not uniform. RT indicates radiotherapy and WBRT, whole-brain radiotherapy. A, Initial brain-directed treatment. B, Treatment received within 6 weeks of initial brain-directed therapy. Treatments received by less than 6 patients during a specific period (ie, BRAF inhibitors and immunotherapy in 2007-2009 and temozolomide in 2013-2016) are not shown. C, Treatment received after 6 weeks of initial brain-directed therapy among patients surviving more than 6 weeks. Treatments received by less than 6 patients during a specific period (ie, surgery in 2010-2012) are not shown.

Shifts were observed in the type of initial brain-directed treatment received over time (Figure 1A). The use of WBRT decreased from 246 patients of 326 patients (75.5%; 95% CI, 70.8%-80.1%) in period 1 to 239 of 460 patients (52.0%; 95% CI, 47.4%-56.5%) in period 3 (*P* < .001). A substantial increase was observed in the number of patients who received conformal radiotherapy (eg, SRS Gamma Knife [Elekta] therapy, ≥3-field intensity-modulated radiotherapy, or volumetric-modulated radiotherapy) as the initial brain-directed therapy, from 11 of 326 patients (3.4%; 95% CI, 1.4%-5.3%) in period 1 to 98 of 460 patients (21.3%; 95% CI, 17.6%-25.0%) in period 3 (*P* < .001). Little change was observed in the use of neurosurgery (69 of 326 patients [21.2%; 95% CI, 16.7%-25.6%] in period 1 to 123 of 460 patients [26.7%; 95% CI, 22.7%-30.8%] in period 3; *P* = .07) (Figure 1A).

Many patients required multiple modalities as part of their initial treatment management, which was defined as the 6-week period measured from the start of the first brain-directed treatment. The proportion of patients receiving WBRT decreased over time (from 294 of 326 patients [90.2%; 95% CI, 87.0%-93.4%] in period 1 to 283 of 460 patients [61.5%; 95% CI, 57.1%-66.0%] in period 3), while the proportion of patients receiving conformal radiotherapy proportionately increased over time (from 14 of 326 patients [4.3%; 95% CI, 2.1%-6.5%] in period 1 to 128 of 460 patients [27.8%; 95% CI, 23.7%-31.9%] in period 3), as did the proportion of patients receiving targeted or immune therapy (from <6 patients [<1.8%; 95% CI, 0.4% to <3.3%] in period 1 to 86 patients [18.7%; 95% CI, 15.1%-22.3%] in period 3; *P* < .001) (Figure 1B).

After initial therapy, many patients required additional brain treatments. The proportion of patients receiving subsequent WBRT increased from 15 patients (4.7%; 95% CI, 2.0%-7.4%) in period 1 to 19 patients (6.3%; 95% CI, 3.2%-9.3%) in period 2 and 49 patients (10.7%; 95% CI, 7.5%-13.9%) in period 3 (*P* = .02). In addition, the proportion of patients receiving subsequent conformal non-WBRT increased from 19 patients (6.0%; 95% CI, 3.0%-9.1%) in period 1 to 58 patients (12.7%; 95% CI, 9.2%-16.1%) in period 3 (*P* = .03). Little change was observed in the number of patients receiving surgery over the 3 periods (10 patients [4.3%; 95% CI, 1.7%-6.9%] in period 1, <6 patients [<2.5%; 95% CI, 0.5% to <4.5%] in period 2, and 19 patients [5.4%; 95% CI, 3.0%-7.7%] in period 3; *P* = .14) (Figure 1C).

**Figure 2.**
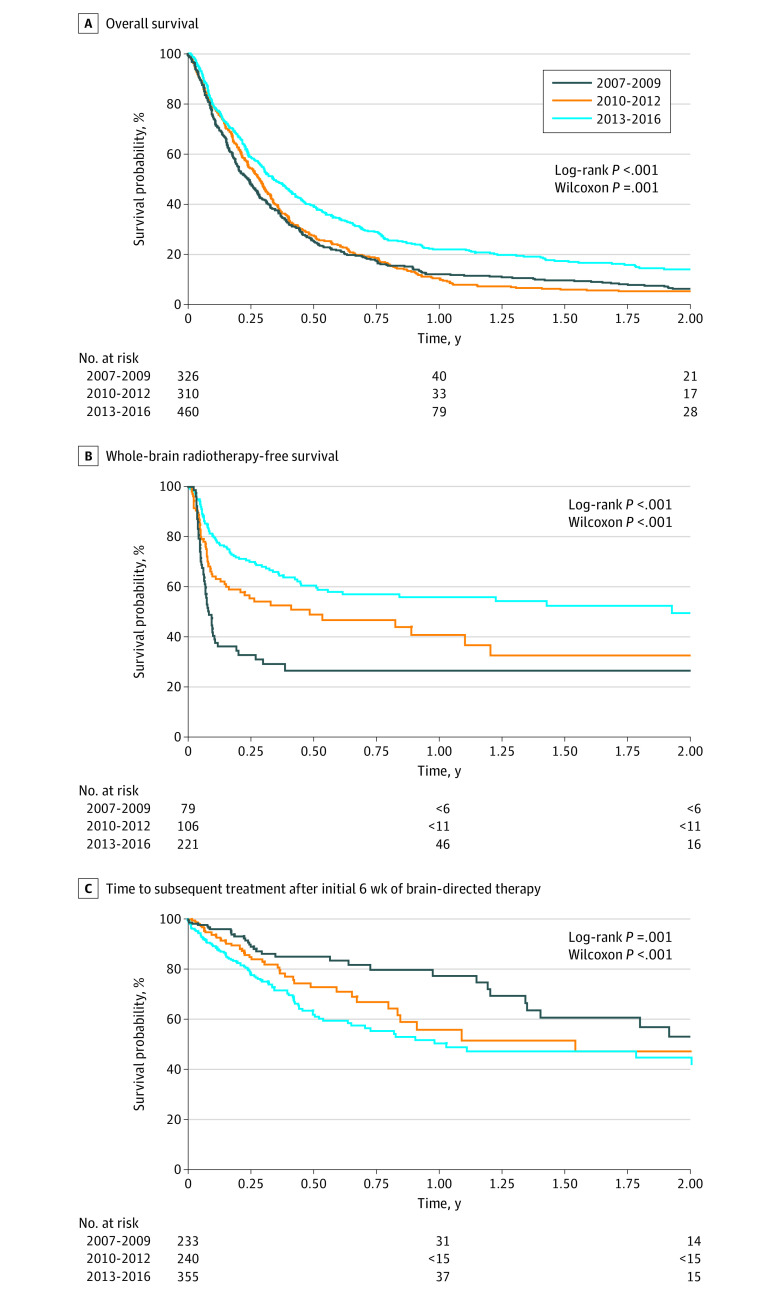
Temporal Trends in Melanoma Brain Metastasis Outcomes In accordance with administrative data privacy regulations, information cannot be reported for groups with 1 to 5 patients. For survival curves, this regulation applies to the number of at-risk patients at specified time points as well as the number of at-risk patients lost between time points. Therefore, the number of at-risk patients were provided only at years 0, 1 and 2. A, Overall survival. Survival from time of first brain-directed treatment (neurosurgery or radiotherapy) for all patients. B, Whole-brain radiotherapy-free survival. Survival among patients who initially received treatment with neurosurgery or conformal radiotherapy. C, Time to subsequent treatment after initial 6 weeks of brain-directed therapy.

Overall survival increased significantly between period 1 and period 3. The 1-year survival probability was 12.3% (95% CI, 9.0%-16.1%) in period 1, 10.7% (95% CI, 7.5%-14.4%) in period 2, and 21.8% (95% CI, 17.9%-25.9%) in period 3. The 2-year survival probability was 6.4% (95% CI, 4.1%-9.5%) in period 1, 5.5% (95% CI, 3.3%-8.4%) in period 2, and 13.8% (95% CI, 10.4%-17.8%) in period 3 (Figure 2A). The adjusted hazard ratio (aHR) of period 3 compared with period 1 was 0.65 (95% CI, 0.56-0.77; *P* < .001). Details of the multivariable model are provided in eTable 1 in the Supplement.

For patients who received localized brain treatment (ie, non-WBRT), we measured WBRT-free survival. In period 1, the 1-year WBRT-free survival probability was 26.7% (95% CI, 16.4%-38.0%), with no further events up to 2 years (Figure 2B). In period 2, the 1-year probability was 40.9% (95% CI, 28.7%-52.8%), and the 2-year probability was 32.7% (95% CI, 19.3%-46.8%). The WBRT-free survival probability further increased in period 3, with 1-year probability at 56.0% (95% CI, 47.8%-63.4%) and 2-year probability at 49.6% (95% CI, 39.4%-59.1%). The aHR for period 3 compared with period 1 was 0.32 (95% CI, 0.22-0.46; *P* < .001) (eTable 2 in the Supplement).

After the initial 6 weeks of brain-directed treatment, during period 1, the 1-year and 2-year probabilities of not receiving further brain-directed therapy were 77.4% (95% CI, 66.5%-85.2%) and 53.2% (95% CI, 36.6%-67.3%), respectively. These probabilities decreased in periods 2 and 3. By period 3, the 1-year probability was 50.5% (95% CI, 41.8%-58.6%), and the 2-year probability was 44.9% (95% CI, 35.0%-54.3%). The multivariable analysis indicated a significantly greater likelihood of receiving additional treatment in period 3 compared with period 1 (aHR, 2.16; 95% CI, 1.48-3.14; *P* < .001) (Figure 2C; eTable 3 in the Supplement).

We hypothesized that the use of magnetic resonance imaging (MRI) for restaging patients with metastatic disease would increase, as would the use of surveillance imaging over time, which would result in earlier detection of brain metastasis in subsequent periods and potential lead-time bias. We found an increased use of MRI over time (Table 3). The results of an adjusted analysis, which accounted for differences in patient-level pretreatment imaging practice, did not alter our conclusion that improvements in survival occurred in period 3 (eTable 4 in the Supplement).

**Table 3.** Temporal Trends of Brain Imaging Performed Within 3 Months Before First Brain-Directed Treatment

Brain imaging	No. (%)										
	Year of first brain treatment										Total
	2007	2008	2009	2010	2011	2012	2013	2014	2015	2016	
Total No.	96	105	125	89	109	112	118	137	144	61	1096
MRI											
Initial	19 (19.8)	20 (19.1)	39 (31.2)	28 (31.5)	29 (26.6)	34 (30.4)	41 (34.8)	48 (35.0)	52 (36.1)	23 (37.7)	333 (30.4)
After CT	35 (36.5)	37 (35.2)	42 (33.6)	35 (39.3)	42 (38.5)	44 (39.3)	42 (35.6)	63 (46.0)	61 (42.4)	27 (44.3)	428 (39.1)
None	42 (43.8)	48 (45.7)	44 (35.2)	26 (29.2)	38 (34.9)	34 (30.4)	35 (29.7)	26 (19.0)	31 (21.5)	11 (18.0)	335 (30.6)

Abbreviations: CT, computed tomography; MRI, magnetic resonance imaging.

We also investigated whether changes in survival were observed when the population was stratified according to the intensity of brain MRI use for surveillance between brain regions. This intensity was defined according to tertile rankings of the 14 health regions in Ontario based on the mean number of brain MRI assessments per patient during the period of 3 to 9 months before the first brain treatment (tertile range, 1-3, with 1 indicating the lowest tertile and 3 indicating the highest tertile of health regions with brain MRI assessments per patient).

The 3 months before the first brain treatment were excluded because imaging use in this period was likely to be partially associated with treatment planning. Significant differences in survival were found for the second and third tertiles (medium and high use of MRI) between periods (eFigure 3 in the Supplement). We performed adjusted analyses that took into account regional surveillance imaging practices (computed tomography and MRI); in all cases, we found improvements in survival between periods that were consistent with the results of the primary analysis (eTable 4 in the Supplement). We also observed that both area-level and patient-level imaging variables suggested better survival among groups with greater use of brain MRI within 3 months of brain treatment. For example, the aHR for patients who received an initial MRI within 3 months of brain treatment was 0.51 (95% CI, 0.43-0.61), and in regions with the highest tertile of MRI use, the aHR was 0.82 (95% CI, 0.70-0.96).

We also performed sensitivity analyses. We considered survival by period among the group of patients whose first palliative treatment was for brain metastasis. This group was considered more homogeneous than the full cohort in terms of the time to detection of brain metastasis, minimizing the potential consequences of lead-time bias. The findings were consistent with the results of the primary analysis (eg, aHR for period 1 vs period 3, 0.69; 95% CI, 0.56-0.84; *P* < .001) (eTable 5 in the Supplement). We also investigated whether the findings were associated with incomplete follow-up of patients in period 3. We limited the analysis to patients who first received treatment between 2007 and 2014. The findings were consistent with the results of the primary analysis (eg, aHR for overall survival in 2013-2014 vs 2007-2009, 0.75; 95% CI, 0.62-0.90; *P* = .002) (eTable 6 in the Supplement).

## Discussion

To our knowledge, this work represents the first population-based study to evaluate the new systemic therapies and radiotherapy techniques specific to patients with melanoma brain metastasis. Our most significant findings were the increase in 1-year survival from 12.3% in period 1 to 21.8% in period 3 and the more than 2-fold increase in 2-year survival from 6.4% in period 1 to 13.8% in period 3. We observed a decreased use of WBRT among patients who initially received focal treatments. These changes over time were associated with a greater use of multiple treatments and a shorter time to subsequent brain treatment in period 3.

The increase in survival is likely associated with multiple factors. First, greater extracranial and intracranial control were likely factors in the increase in overall survival. Checkpoint and BRAF inhibitors have been reported to have activity in brain metastases.^11,12,19,20,21^ In single-arm prospective studies, these therapeutic agents have been found to have intracranial response rates that range from 5% to 39%.^11,12,19,20,21^ The CheckMate 204 study^22^ investigated the use of a combination of nivolumab and ipilimumab therapies for the treatment of patients with no neurological symptoms and at least 1 nonirradiated brain metastasis, reporting that the rate of intracranial benefit was 57%, with a complete response in 26% of patients. A retrospective study indicated that combining stereotactic radiosurgery with immunotherapy was associated with greater intracranial control.^23^

The observed increase in overall survival may have been owing in part to lead-time bias or changes in the case mix over time, although our adjusted analyses did not support this conclusion. We also note that improvements in survival followed patterns similar to those reported in a larger population-based cohort study from Ontario that included patients with metastatic or unresectable melanoma with or without brain metastasis.^18^ We also observed that the time from the first diagnosis of any stage of melanoma to the first palliative treatment was stable between periods. The time between the first palliative treatment and the first local brain treatment increased over time, which would bias the results toward an underestimate of any survival benefit between periods. The improvements in survival observed in period 3 remained unchanged after adjustment for this time difference.

We also observed an increase in the annual number of patients with brain metastases and an increase in the overall incidence of melanoma in Ontario over time (in 2007, 1909 melanoma incidence cases, 96 with initial brain-directed treatment; in 2015, 2437 melanoma incidence cases, 144 with initial brain-directed treatment).^18^ We hypothesized that much of the increase in the proportion of patients with brain metastasis was associated with the earlier and more frequent detection of brain metastases, many of which would otherwise be asymptomatic. We were not able to directly adjust for changes in the case mix over time. For this reason, lead-time bias in the identification of earlier asymptomatic disease in period 3 cannot be excluded as a factor in the survival improvement observed. However, we adjusted for changes in imaging practice, which were associated with survival differences. The survival improvement in period 3 that we observed in our primary analysis remained unchanged in these adjusted analyses.

The purpose of our analyses of brain imaging data was to investigate and control for potential lead-time bias. However, it is noteworthy that both area-level and patient-level imaging variables indicated better survival among groups who had greater use of advanced imaging techniques in the form of brain MRIs. For example, the aHR for patients who received an initial MRI within 3 months of brain treatment was 0.51; in regions with the highest tertile of MRI use, the aHR was 0.82 (eTable 4 in the Supplement). Our analyses raise the question of whether the use of advanced imaging techniques provides a survival benefit for patients by identifying brain metastasis when the disease burden is lower, with potentially less serious clinical implications. Our findings cannot in themselves confirm this hypothesis.

We observed substantial changes in the treatment management of melanoma brain metastasis over time. However, 61.5% of patients continued to receive WBRT in period 3. This continuation may be associated with a number of factors, including substantial disease burden at the time the brain metastasis was first detected and geographic accessibility to focal brain therapies. The disease burden at the time of metastasis detection could possibly be improved by optimizing the use of brain MRI among patients with melanoma. Geographic accessibility has been reported to be an important factor in Ontario, and cancer centers with on-site SRS programs have an increased likelihood of using SRS.^24^ During the period of our study, only selected centers in Ontario performed stereotactic brain treatments, although more centers now offer local therapies.

In a recent study, Hong et al^25^ reported the results of a randomized clinical trial of WBRT compared with observation after surgery and/or SRS among patients with 1 to 3 melanoma brain metastases. The study did not find an association with its primary end point of distant intracranial failure. This study provided further data to suggest that administering local therapy without initial WBRT for the treatment of many patients with melanoma brain metastasis is appropriate and highlighted the need to optimize the use of local and systemic therapies to treat melanoma brain metastasis in patients in Ontario and other jurisdictions. The study also added to the findings of the Aoyama et al^1^ study and the European Organisation for Research and Treatment of Cancer (EORTC) 22952-26001^4^ clinical trial, in which no overall survival difference was detected in patients who received WBRT in addition to either surgery or SRS. Studies have also indicated that the addition of WBRT to SRS in patients with limited intracranial disease is associated with further neurocognitive decline.^2,26^

### Limitations

This study has several limitations. The retrospective nature of the administrative data is noted. Notably, brain imaging practice changed over time, which likely resulted in greater detection of small and asymptomatic brain metastases. Using imaging claims data, we were able to investigate the potential association of this factor with survival outcomes. All analyses indicated an association with improvements in survival, despite this change in practice. Our study focused only on patients who received local treatment with either radiotherapy or surgery. The analysis did not account for patients who received treatment with supportive care or systemic therapy alone. We note that the latest cohort in our study was from the 2013 to 2016 period, which predated the 2018 publication of combination immunotherapy results for melanoma brain metastasis without previous local treatment.^22,27^ We note that the radiotherapy data did not allow us to exclude patients receiving cranial radiotherapy secondary to leptomeningeal carcinomatosis. We acknowledge that results from our analyses cannot in themselves separate the factors of systemic therapy and radiotherapy at the population level. As with most studies that use administrative data, our study did not have access to the patient’s intracranial or extracranial disease status and, for this reason, we conducted a period-based comparison.

## Conclusions

The advent of new radiotherapy treatment modalities and novel systemic treatments for melanoma brain metastasis was associated with increased survival and decreased use of WBRT, although the decrease in WBRT use resulted in more frequent brain treatment courses. In the cohort from period 3 (2013 to 2016), more than half of the patients with melanoma brain metastasis continued to receive WBRT in routine practice, and many did not receive targeted therapeutic agents or immunotherapies. A risk of lead-time bias existed because of the earlier detection of brain metastasis in later periods, although the adjusted analyses were consistent with the findings of our primary analyses. The findings suggest important benefits for people with melanoma brain metastasis, with opportunities to further improve outcomes by optimizing the use of advanced radiotherapy techniques and novel systemic therapies.

## Notes

Supplement. **eTable 1.** Multivariable Analysis for Overall Survival **eTable 2.** Multivariable Analysis for Whole-Brain Radiotherapy-Free Survival **eTable 3.** Multivariable Analysis for Time to Subsequent Brain Treatment **eTable 4.** Multivariable Analysis for Overall Survival Adjusted for Imaging Practice Differences Before First Brain-Directed Treatment **eTable 5.** Sensitivity Analysis Among Patients Whose First Palliative Treatment Was Brain-Directed (Surgery or Radiotherapy): Characteristics and Multivariable Analysis for Overall Survival **eTable 6.** Sensitivity Analysis Among Patients Treated From January 1, 2007, to December 31, 2014: Multivariable Analysis for Overall Survival **eFigure 1.** Timeline for Changes in Evidence and Recommendations Relevant to Melanoma Brain Metastasis Systemic Therapy and Radiotherapy **eFigure 2.** Identification of Melanoma Patients Treated Locally for Brain Metastases With Radiotherapy or Surgery in Ontario From January 1, 2007, to June 30, 2016 **eFigure 3.** Overall Survival According to Period, Stratified by the Tertiles of Mean Number of Brain MRI Assessments per Patient by LHIN Health Region in the 3 to 9 Months Before the First Brain Treatment
