# Supplementary material for: Association of Innovations in Radiotherapy and Systemic Treatments With Clinical Outcomes in Patients With Melanoma Brain Metastasis From 2007 to 2016
Source: JAMA Netw Open. 2020 Jul 6;3(7):e208204. doi: 10.1001/jamanetworkopen.2020.8204 (PMC7339137; doi:10.1001/jamanetworkopen.2020.8204)
Supplement: Supplement. — eTable 1. Multivariable Analysis for Overall Survival eTable 2. Multivariable Analysis for Whole-Brain Radiotherapy-Free Survival eTable 3. Multivariable Analysis for Time to Subsequent Brain Treatment eTable 4. Multivariable Analysis for Overall Survival Adjusted for Imaging Practice Differences Before First Brain-Directed Treatment eTable 5. Sensitivity Analysis Among Patients Whose First Palliative Treatment Was Brain-Directed (Surgery or Radiotherapy): Characteristics and Multivariable Analysis for Overall Survival eTable 6. Sensitivity Analysis Among Patients Treated From January 1, 2007, to December 31, 2014: Multivariable Analysis for Overall Survival eFigure 1. Timeline for Changes in Evidence and Recommendations Relevant to Melanoma Brain Metastasis Systemic Therapy and Radiotherapy eFigure 2. Identification of Melanoma Patients Treated Locally for Brain Metastases With Radiotherapy or Surgery in Ontario From January 1, 2007, to June 30, 2016 eFigure 3. Overall Survival According to Period, Stratified by the Tertiles of Mean Number of Brain MRI Assessments per Patient by LHIN Health Region in the 3 to 9 Months Before the First Brain Treatment [file jamanetwopen-3-e208204-s001.pdf]

Supplementary Online Content

Brastianos HC, Nguyen P, Sahgal A, Eisenhauer EA, Baetz T, Hanna TP. Association of innovations in radiotherapy and systemic treatments with clinical outcomes in patients with melanoma brain metastasis from 2007 to 2016. *JAMA Netw Open*. 2020;3(7):e208204. doi:10.1001/jamanetworkopen.2020.8204

eTable 1. Multivariable Analysis for Overall Survival

eTable 2. Multivariable Analysis for Whole-Brain Radiotherapy-Free Survival

eTable 3. Multivariable Analysis for Time to Subsequent Brain Treatment

eTable 4. Multivariable Analysis for Overall Survival Adjusted for Imaging Practice Differences Before First Brain-Directed Treatment

eTable 5. Sensitivity Analysis Among Patients Whose First Palliative Treatment Was Brain-Directed (Surgery or Radiotherapy): Characteristics and Multivariable Analysis for Overall Survival

eTable 6. Sensitivity Analysis Among Patients Treated From January 1, 2007, to December 31, 2014: Multivariable Analysis for Overall Survival

eFigure 1. Timeline for Changes in Evidence and Recommendations Relevant to Melanoma Brain Metastasis Systemic Therapy and Radiotherapy

eFigure 2. Identification of Melanoma Patients Treated Locally for Brain Metastases With Radiotherapy or Surgery in Ontario From January 1, 2007, to June 30, 2016

eFigure 3. Overall Survival According to Period, Stratified by the Tertiles of Mean Number of Brain MRI Assessments per Patient by LHIN Health Region in the 3 to 9 Months Before the First Brain Treatment

This supplementary material has been provided by the authors to give readers additional information about their work.

© 2020 Brastianos HC et al. *JAMA Network Open*.

eTable 1. Multivariable Analysis for Overall Survival

	Full Model		Stepwise Model[1]	
Patient Characteristic	HR (95% CI)	P-value	HR (95% CI)	P-value
Year of first brain-directed treatment				
2007-2009	Reference		Reference	
2010-2012	0.96 (0.81-1.12)	0.575	0.93 (0.80-1.10)	0.401
2013-2016	0.64 (0.55-0.76)	<0.001	0.65 (0.56-0.77)	<0.001
Age				
20-39	Reference		Reference	
40-49	0.88 (0.65-1.20)	0.422	0.88 (0.65-1.21)	0.445
50-59	0.92 (0.69-1.24)	0.596	0.93 (0.69-1.25)	0.612
60-69	1.13 (0.84-1.52)	0.425	1.15 (0.86-1.54)	0.357
70-79	1.41 (1.04-1.93)	0.029	1.45 (1.07-1.97)	0.018
80+	2.05 (1.48-2.84)	<0.001	2.11 (1.52-2.94)	<0.001
Sex				
Female	Reference			
Male	1.04 (0.91-1.19)	0.559		
Elixhauser comorbidity index				
0	Reference		Reference	
1	1.34 (1.14-1.56)	<0.001	1.33 (1.14-1.55)	<0.001
2	1.15 (0.94-1.42)	0.177	1.13 (0.92-1.40)	0.241
3+	1.37 (1.10-1.71)	0.005	1.37 (1.10-1.70)	0.004
Neighbourhood income quintile				
1	Reference		Reference	
2	0.83 (0.68-1.01)	0.060	0.82 (0.67-0.99)	0.042
3	0.75 (0.60-0.94)	0.011	0.74 (0.59-0.92)	0.007
4	0.87 (0.72-1.06)	0.159	0.87 (0.72-1.05)	0.137
5	0.78 (0.64-0.95)	0.013	0.77 (0.63-0.93)	0.007
Rurality Index for Ontario (RIO)				
Urban (RIO<10)	0.87 (0.70-1.09)	0.224		
Suburban (10≤RIO<40)	0.89 (0.70-1.13)	0.331		
Rural (40≤RIO)	Reference			
Morphology of first melanoma diagnosis				
Nodular	1.13 (0.96-1.35)	0.147		
Superficial spreading	1.06 (0.88-1.27)	0.554		
Acral lentiginous, desmoplastic, lentigo maligna, other, or multiple primaries	1.10 (0.87-1.38)	0.434		
Not otherwise specified	Reference			
Topography of first melanoma diagnosis				
External ear	1.38 (0.98-1.93)	0.065	1.45 (1.05-2.00)	0.025
Lower limb and hip	1.20 (0.95-1.51)	0.125	1.18 (0.96-1.46)	0.118
Scalp and neck	1.21 (0.90-1.63)	0.207	1.24 (0.93-1.64)	0.142
Trunk	1.32 (1.07-1.64)	0.010	1.32 (1.10-1.58)	0.003
Upper limb and shoulder	1.53 (1.20-1.96)	0.001	1.51 (1.21-1.89)	<0.001
Other parts of face	1.02 (0.72-1.44)	0.919	1.05 (0.76-1.45)	0.785
Malignant neoplasm of skin, site unspecified; or multiple primaries	Reference		Reference	
Continued:				

© 2020 Brastianos HC et al. *JAMA Network Open*.

Continued:	**Full Model**		**Stepwise Model**[1]	
	HR (95% CI)	P-value	HR (95% CI)	P-value
Time between first melanoma diagnosis and first palliative treatment				
0-5 months	Reference			
6-11 months	1.15 (0.89-1.49)	0.292		
12-17 months	0.95 (0.73-1.24)	0.713		
18-23 months	0.93 (0.71-1.22)	0.600		
24-35 months	0.95 (0.75-1.20)	0.685		
36-59 months	1.04 (0.82-1.33)	0.727		
60-119 months	0.78 (0.61-1.00)	0.048		
120+ months	0.93 (0.71-1.20)	0.565		
Time between first palliative treatment and first brain-directed treatment				
At first palliative treatment	Reference		Reference	
0-2 months	1.55 (1.21-1.99)	0.001	1.59 (1.24-2.04)	<0.001
3-5 months	1.30 (1.01-1.68)	0.041	1.35 (1.05-1.73)	0.018
6-11 months	1.13 (0.88-1.44)	0.339	1.17 (0.92-1.49)	0.196
12+ months	1.06 (0.87-1.31)	0.559	1.07 (0.87-1.31)	0.527
Global Proportionality Test		0.018		0.085

Notes:
1. In the stepwise model, a significance level of 0.20 was used for model selection and variables with a significance level of 0.10 remained in the final model
HR = hazard ratio, CI = confidence interval

© 2020 Brastianos HC et al. *JAMA Network Open*.

eTable 2. Multivariable Analysis for Whole-Brain Radiotherapy-Free Survival

	Full Model		Stepwise Model[1]	
Patient Characteristic	HR (95% CI)	P-value	HR (95% CI)	P-value
Year of first brain-directed treatment				
2007-2009	Reference		Reference	
2010-2012	0.66 (0.43-1.02)	0.062	0.58 (0.39-0.86)	0.007
2013-2016	0.37 (0.25-0.56)	<0.001	0.32 (0.22-0.46)	<0.001
Age				
20-39	Reference		Reference	
40-49	1.55 (0.76-3.15)	0.229	1.19 (0.62-2.30)	0.603
50-59	1.68 (0.86-3.27)	0.126	1.44 (0.77-2.68)	0.255
60-69	2.02 (1.03-3.97)	0.042	1.72 (0.94-3.16)	0.079
70-79	1.77 (0.86-3.63)	0.123	1.54 (0.82-2.90)	0.177
80+	0.51 (0.18-1.44)	0.202	0.55 (0.21-1.44)	0.224
Sex				
Female	Reference			
Male	0.89 (0.64-1.24)	0.491		
Elixhauser comorbidity index				
0	Reference			
1	1.10 (0.74-1.62)	0.636		
2	1.50 (0.92-2.47)	0.106		
3+	0.85 (0.47-1.54)	0.598		
Neighbourhood income quintile				
1	Reference			
2	1.32 (0.78-2.23)	0.296		
3	1.08 (0.64-1.83)	0.776		
4	1.05 (0.61-1.81)	0.850		
5	0.78 (0.45-1.37)	0.393		
Rurality Index for Ontario (RIO)				
Urban (RIO<10)	0.91 (0.57-1.46)	0.706		
Suburban (10≤RIO<40)	0.74 (0.44-1.26)	0.264		
Rural (40≤RIO)	Reference			
Morphology of first melanoma diagnosis				
Nodular	1.02 (0.67-1.55)	0.940		
Superficial spreading	1.10 (0.68-1.80)	0.697		
Acral lentiginous, desmoplastic, lentigo maligna, other, or multiple primaries	1.23 (0.70-2.16)	0.479		
Not otherwise specified	Reference			
Topography of first melanoma diagnosis				
External ear	0.99 (0.32-3.04)	0.981		
Lower limb and hip	0.53 (0.28-0.99)	0.045		
Scalp and neck	0.48 (0.25-0.93)	0.029		
Trunk	0.65 (0.40-1.05)	0.077		
Upper limb and shoulder	0.70 (0.40-1.22)	0.209		
Other parts of face	0.74 (0.37-1.51)	0.413		
Malignant neoplasm of skin, site unspecified; or multiple primaries	Reference			
Continued:				

© 2020 Brastianos HC et al. *JAMA Network Open*.

Continued:	**Full Model**		**Stepwise Model[1]**	
	HR (95% CI)	P-value	HR (95% CI)	P-value
Time between first melanoma diagnosis and first palliative treatment				
0-5 months	Reference			
6-11 months	1.01 (0.49-2.11)	0.973		
12-17 months	0.98 (0.52-1.86)	0.946		
18-23 months	1.03 (0.51-2.06)	0.939		
24-35 months	1.69 (0.99-2.88)	0.057		
36-59 months	1.53 (0.86-2.72)	0.147		
60-119 months	1.01 (0.57-1.78)	0.975		
120+ months	1.34 (0.68-2.65)	0.397		
Time between first brain-directed treatment and first palliative treatment				
At first palliative treatment	Reference			
0-2 months	0.58 (0.23-1.43)	0.233		
3-5 months	0.49 (0.23-1.05)	0.065		
6-11 months	0.57 (0.31-1.06)	0.077		
12+ months	0.94 (0.59-1.49)	0.776		
Global Proportionality Test		0.762		0.301

Notes:
1. In the stepwise model, a significance level of 0.20 was used for model selection and variables with a significance level of 0.10 remained in the final model
HR = hazard ratio, CI = confidence interval

© 2020 Brastianos HC et al. *JAMA Network Open*.

eTable 3. Multivariable Analysis for Time to Subsequent Brain Treatment

	**Full Model**		**Stepwise Model[1]**	
Patient Characteristic	HR (95% CI)	P-value	HR (95% CI)	P-value
Year of first brain-directed treatment				
2007-2009	Reference		Reference	
2010-2012	1.50 (0.95-2.36)	0.080	1.59 (1.04-2.42)	0.033
2013-2016	2.02 (1.35-3.04)	0.001	2.16 (1.48-3.14)	<0.001
Age				
20-39	Reference		Reference	
40-49	1.30 (0.71-2.36)	0.393	1.06 (0.61-1.87)	0.829
50-59	1.08 (0.60-1.95)	0.800	0.90 (0.53-1.53)	0.685
60-69	0.87 (0.48-1.59)	0.657	0.73 (0.42-1.28)	0.275
70-79	1.17 (0.60-2.28)	0.654	0.95 (0.52-1.72)	0.858
80+	0.17 (0.04-0.66)	0.010	0.15 (0.04-0.58)	0.007
Sex				
Female	Reference			
Male	0.99 (0.71-1.37)	0.933		
Elixhauser comorbidity index				
0	Reference			
1	0.92 (0.61-1.38)	0.680		
2	0.84 (0.46-1.53)	0.569		
3+	1.11 (0.61-2.02)	0.741		
Neighbourhood income quintile				
1	Reference			
2	0.83 (0.51-1.37)	0.467		
3	0.64 (0.38-1.08)	0.096		
4	0.88 (0.53-1.45)	0.620		
5	0.73 (0.45-1.18)	0.201		
Rurality Index for Ontario (RIO)				
Urban (RIO<10)	0.82 (0.49-1.36)	0.434	0.85 (0.52-1.37)	0.496
Suburban (10≤RIO<40)	0.45 (0.25-0.80)	0.007	0.46 (0.26-0.80)	0.007
Rural (40≤RIO)	Reference		Reference	
Morphology of first melanoma diagnosis				
Nodular	1.31 (0.87-1.97)	0.192		
Superficial spreading	1.13 (0.69-1.85)	0.633		
Acral lentiginous, desmoplastic, lentigo maligna, other, or multiple primaries	0.92 (0.53-1.61)	0.775		
Not otherwise specified	Reference			
Topography of first melanoma diagnosis				
External ear	0.99 (0.41-2.40)	0.983		
Lower limb and hip	0.65 (0.36-1.18)	0.157		
Scalp and neck	0.71 (0.36-1.42)	0.338		
Trunk	0.70 (0.43-1.16)	0.167		
Upper limb and shoulder	1.10 (0.63-1.93)	0.734		
Other parts of face	1.26 (0.59-2.68)	0.553		
Malignant neoplasm of skin, site unspecified; or multiple primaries	Reference			
Continued:				

© 2020 Brastianos HC et al. *JAMA Network Open*.

Continued:	**Full Model**		**Stepwise Model[1]**	
	HR (95% CI)	P-value	HR (95% CI)	P-value
Time between first melanoma diagnosis and first palliative treatment				
0-5 months	Reference			
6-11 months	1.64 (0.84-3.23)	0.150		
12-17 months	1.63 (0.92-2.87)	0.094		
18-23 months	1.95 (1.08-3.53)	0.027		
24-35 months	1.44 (0.77-2.70)	0.251		
36-59 months	1.16 (0.61-2.22)	0.648		
60-119 months	1.05 (0.59-1.85)	0.878		
120+ months	1.30 (0.67-2.54)	0.435		
Time between first palliative treatment and first brain-directed treatment				
At first palliative treatment	Reference			
0-2 months	1.09 (0.59-2.04)	0.776		
3-5 months	0.94 (0.52-1.68)	0.830		
6-11 months	0.89 (0.52-1.52)	0.666		
12+ months	1.39 (0.82-2.38)	0.224		
Global Proportionality Test		0.247		0.176

Notes:
1. In the stepwise model, a significance level of 0.20 was used for model selection and variables with a significance level of 0.10 remained in the final model
HR = hazard ratio, CI = confidence interval

© 2020 Brastianos HC et al. *JAMA Network Open*.

eTable 4. Multivariable Analysis for Overall Survival Adjusted for Imaging Practice Differences Before First Brain-Directed Treatment

	Full Model[1] with Brain Imaging Practices within 3 Months prior to First Brain Treatment				Full Model[1] with Brain Imaging Practices within 3-9 Months prior to First Brain Treatment							
	Brain MRI		Brain Imaging Type		Brain CT		Brain MRI		Brain CT or MRI		Brain Imaging Type	
	HR (95% CI)	P-value	HR (95% CI)	P-value	HR (95% CI)	P-value	HR (95% CI)	P-value	HR (95% CI)	P-value	HR (95% CI)	P-value
Year of first brain-directed treatment												
2007-2009	Reference		Reference		Reference		Reference		Reference		Reference	
2010-2012	0.93 (0.79-1.10)	0.403	1.00 (0.86-1.18)	0.975	0.97 (0.83-1.14)	0.703	0.94 (0.80-1.11)	0.452	0.96 (0.82-1.13)	0.636	0.96 (0.82-1.13)	0.607
2013-2016	0.63 (0.53-0.75)	<0.001	0.71 (0.60-0.84)	<0.001	0.66 (0.55-0.78)	<0.001	0.63 (0.54-0.75)	<0.001	0.66 (0.56-0.78)	<0.001	0.65 (0.55-0.76)	<0.001
Regional brain MRI use[2]												
1st tertile	Reference											
2nd tertile	0.82 (0.69-0.99)	0.037										
3rd tertile	0.82 (0.70-0.96)	0.015										
Type of brain imaging at patient level[3]												
No MRI			Reference									
Initial MRI			0.51 (0.43-0.61)	<0.001								
Subsequent MRI after CT			0.57 (0.49-0.66)	<0.001								
Regional brain CT use[4]												
1st tertile					Reference							
2nd tertile					1.02 (0.86-1.21)	0.817						
3rd tertile					1.09 (0.92-1.29)	0.330						
Regional brain MRI use[5]												
1st tertile							Reference					
2nd tertile							0.84 (0.71-0.99)	0.036				
3rd tertile							0.92 (0.78-1.09)	0.337				
Regional brain CT or MRI use[6]												
1st tertile									Reference			
2nd tertile									1.13 (0.96-1.34)	0.134		
3rd tertile									1.07 (0.92-1.25)	0.361		
Type of brain imaging at patient level[7]												
No imaging											Reference	
CT without MRI											0.99 (0.78-1.25)	0.927
MRI with or without CT											0.94 (0.77-1.14)	0.517
Interaction with year of first brain treatment and imaging practices[8]	N/A	0.428	N/A	0.222	N/A	0.385	N/A	0.074	N/A	0.280	N/A	0.327

Notes:

1. Full model adjusted for year of first brain-directed treatment, age, sex, Elixhauser comorbidity index, neighbourhood income quintiles, Rurality Index for Ontario, morphology and topography of first melanoma diagnosis, time between first melanoma diagnosis and first palliative treatment, time between first brain-directed and first palliative treatments, and brain imaging assessment
2. Tertiles were based on the proportion of patients receiving ≥1 brain MRI assessment by LHIN health region treatment
3. Type of brain imaging was based on any patient-level brain CT or MRI assessment
4. Tertiles were based on the proportion of patients receiving ≥1 brain CT assessment by LHIN health region treatment
5. Tertiles were based on the mean number of brain MRI assessments per patient by LHIN health region
6. Tertiles were based on the proportion of patients receiving ≥1 brain CT or MRI assessment by LHIN health region
7. Type of brain imaging was based on any patient-level brain CT or MRI assessment
8. Interaction effects were assessed with joint tests under full-rank parameterization. Based on results, interaction effects were not included in the full model.

HR = hazard ratio, CI = confidence interval

© 2020 Brastianos HC et al. *JAMA Network Open.*

eTable 5. Sensitivity Analysis Among Patients Whose First Palliative Treatment Was Brain-Directed (Surgery or Radiotherapy): Characteristics and Multivariable Analysis for Overall Survival

A. Characteristics

Patient Characteristic	Total (N=713)
Age	
Mean ± SD	62.61 ± 13.97
Median (IQR)	62 (53-74)
Age (categorized)	
20-39	42 (5.89%)
40-49	87 (12.20%)
50-59	168 (23.56%)
60-69	168 (23.56%)
70-79	162 (22.72%)
80+	86 (12.06%)
Sex	
Female	214 (30.01%)
Male	499 (69.99%)
Elixhauser comorbidity index	
0	397 (55.68%)
1	154 (21.60%)
2	79 (11.08%)
3+	83 (11.64%)
Neighbourhood income quintile	
Missing, or 1	122 (17.11%)
2	135 (18.93%)
3	127 (17.81%)
4	169 (23.70%)
5	160 (22.44%)
Rurality Index for Ontario (RIO)	
Urban (RIO<10)	448 (62.83%)
Suburban (10≤RIO<40)	194 (27.21%)
Missing, or rural (40≤RIO)	71 (9.96%)
Morphology of first melanoma diagnosis	
Nodular	161 (22.58%)
Superficial spreading	120 (16.83%)
Acral lentiginous, desmoplastic, lentigo maligna, other, or multiple primaries	73 (10.24%)
Not otherwise specified	359 (50.35%)
Topography of first melanoma diagnosis	
External ear	22 (3.09%)
Lower limb and hip	88 (12.34%)
Scalp and neck	57 (7.99%)
Trunk	230 (32.26%)
Upper limb and shoulder	121 (16.97%)
Other parts of face	44 (6.17%)
Malignant neoplasm of skin, site unspecified; or multiple primaries	151 (21.18%)

© 2020 Brastianos HC et al. *JAMA Network Open.*

Continued:	
Patient Characteristic (continued)	Total (N=713)
Time (months) between first melanoma diagnosis and first palliative treatment	
Mean ± SD	45.75 ± 63.10
Median (IQR)	24 (4-58)
Time (categorized) between first melanoma diagnosis and first palliative treatment	
0-5 months	189 (26.51%)
6-11 months	48 (6.73%)
12-17 months	68 (9.54%)
18-23 months	48 (6.73%)
24-35 months	87 (12.20%)
36-59 months	100 (14.03%)
60-119 months	107 (15.01%)
120+ months	66 (9.26%)

B. Multivariable analysis for overall survival

	Full Model		Stepwise Model[1]	
Patient Characteristic	HR (95% CI)	P-value	HR (95% CI)	P-value
Year of first brain-directed treatment				
2007-2009	Reference		Reference	
2010-2012	0.91 (0.75-1.11)	0.367	0.89 (0.74-1.07)	0.224
2013-2016	0.69 (0.56-0.84)	<0.001	0.69 (0.56-0.84)	<0.001
Age				
20-39	Reference		Reference	
40-49	0.95 (0.65-1.37)	0.768	0.87 (0.61-1.25)	0.456
50-59	0.96 (0.68-1.36)	0.829	0.92 (0.67-1.27)	0.615
60-69	1.23 (0.86-1.75)	0.253	1.15 (0.83-1.59)	0.396
70-79	1.52 (1.05-2.18)	0.025	1.43 (1.01-2.01)	0.042
80+	2.08 (1.41-3.06)	<0.001	1.97 (1.35-2.87)	<0.001
Sex				
Female	Reference			
Male	1.08 (0.90-1.29)	0.407		
Elixhauser comorbidity index				
0	Reference		Reference	
1	1.40 (1.15-1.71)	0.001	1.40 (1.15-1.70)	0.001
2	1.26 (0.97-1.64)	0.087	1.36 (1.05-1.75)	0.018
3+	1.48 (1.13-1.95)	0.005	1.59 (1.22-2.06)	0.001
Neighbourhood income quintile				
1	Reference			
2	0.94 (0.74-1.19)	0.611		
3	0.77 (0.58-1.01)	0.057		
4	0.80 (0.63-1.02)	0.069		
5	0.81 (0.63-1.04)	0.099		
Rurality Index for Ontario (RIO)				
Urban (RIO<10)	0.89 (0.67-1.18)	0.422		
Suburban (10≤RIO<40)	0.79 (0.58-1.09)	0.147		
Rural (40≤RIO)	Reference			
Continued:				

© 2020 Brastianos HC et al. *JAMA Network Open*.

Continued:	**Full Model**		**Stepwise Model[1]**	
	HR (95% CI)	P-value	HR (95% CI)	P-value
Morphology of first melanoma diagnosis				
Nodular	1.16 (0.94-1.44)	0.178		
Superficial spreading	0.97 (0.77-1.22)	0.784		
Acral lentiginous, desmoplastic, lentigo maligna, other, or multiple primaries	1.14 (0.86-1.52)	0.352		
Not otherwise specified	Reference			
Topography of first melanoma diagnosis				
External ear	1.26 (0.83-1.93)	0.282	1.41 (0.94-2.13)	0.098
Lower limb and hip	1.16 (0.84-1.59)	0.364	1.23 (0.94-1.62)	0.135
Scalp and neck	1.25 (0.87-1.79)	0.220	1.27 (0.91-1.78)	0.161
Trunk	1.36 (1.04-1.78)	0.027	1.42 (1.13-1.77)	0.003
Upper limb and shoulder	1.58 (1.18-2.11)	0.002	1.59 (1.22-2.07)	0.001
Other parts of face	1.30 (0.88-1.91)	0.190	1.42 (0.99-2.03)	0.054
Malignant neoplasm of skin, site unspecified; or multiple primaries	Reference		Reference	
Time between first melanoma diagnosis and first palliative treatment				
0-5 months	Reference			
6-11 months	1.22 (0.85-1.73)	0.279		
12-17 months	0.84 (0.60-1.18)	0.319		
18-23 months	1.02 (0.71-1.47)	0.901		
24-35 months	0.97 (0.72-1.29)	0.815		
36-59 months	1.19 (0.89-1.60)	0.234		
60-119 months	0.86 (0.63-1.15)	0.306		
120+ months	1.00 (0.71-1.40)	0.977		
Global Proportionality Test		0.367		0.215

Notes:

1. In the stepwise model, a significance level of 0.20 was used for model selection and variables with a significance level of 0.10 remained in the final model

HR = hazard ratio, CI = confidence interval

© 2020 Brastianos HC et al. *JAMA Network Open*.

eTable 6. Sensitivity Analysis Among Patients Treated From January 1, 2007, to December 31, 2014: Multivariable Analysis for Overall Survival

	**Full Model**		**Stepwise Model[1]**	
Patient Characteristic	HR (95% CI)	P-value	HR (95% CI)	P-value
Year of first brain-directed treatment				
2007-2009	Reference		Reference	
2010-2012	0.97 (0.83-1.14)	0.733	0.97 (0.83-1.13)	0.678
2013-2014	0.74 (0.61-0.90)	0.002	0.75 (0.62-0.90)	0.002
Age				
20-39	Reference		Reference	
40-49	0.86 (0.63-1.18)	0.358	0.88 (0.64-1.20)	0.407
50-59	0.94 (0.69-1.28)	0.698	0.97 (0.71-1.32)	0.830
60-69	1.06 (0.78-1.43)	0.729	1.08 (0.79-1.46)	0.636
70-79	1.57 (1.13-2.18)	0.008	1.62 (1.17-2.25)	0.004
80+	2.04 (1.46-2.86)	<0.001	2.11 (1.50-2.97)	<0.001
Sex				
Female	Reference			
Male	1.06 (0.92-1.22)	0.443		
Elixhauser comorbidity index				
0	Reference		Reference	
1	1.29 (1.09-1.53)	0.004	1.29 (1.09-1.52)	0.003
2	1.32 (1.06-1.64)	0.013	1.32 (1.06-1.63)	0.013
3+	1.47 (1.15-1.87)	0.002	1.45 (1.14-1.85)	0.003
Neighbourhood income quintile				
1	Reference		Reference	
2	0.74 (0.60-0.92)	0.006	0.73 (0.59-0.91)	0.004
3	0.65 (0.51-0.83)	0.001	0.65 (0.51-0.83)	0.001
4	0.78 (0.64-0.96)	0.021	0.77 (0.63-0.95)	0.014
5	0.78 (0.63-0.97)	0.022	0.77 (0.62-0.94)	0.012
Rurality Index for Ontario (RIO)				
Urban (RIO<10)	0.93 (0.73-1.19)	0.568		
Suburban (10≤RIO<40)	0.90 (0.69-1.18)	0.449		
Rural (40≤RIO)	Reference			
Morphology of first melanoma diagnosis				
Nodular	1.10 (0.91-1.32)	0.334		
Superficial spreading	1.03 (0.84-1.25)	0.809		
Acral lentiginous, desmoplastic, lentigo maligna, other, or multiple primaries	1.08 (0.85-1.37)	0.538		
Not otherwise specified	Reference			
Topography of first melanoma diagnosis				
External ear	1.37 (0.95-1.97)	0.096	1.43 (1.00-2.05)	0.052
Lower limb and hip	1.26 (0.99-1.59)	0.062	1.28 (1.01-1.61)	0.042
Scalp and neck	1.17 (0.84-1.62)	0.346	1.21 (0.88-1.66)	0.254
Trunk	1.36 (1.08-1.70)	0.009	1.40 (1.13-1.74)	0.002
Upper limb and shoulder	1.45 (1.11-1.88)	0.006	1.47 (1.14-1.90)	0.004
Other parts of face	1.21 (0.85-1.72)	0.294	1.25 (0.88-1.78)	0.206
Malignant neoplasm of skin, site unspecified; or multiple primaries	Reference		Reference	
Continued:				

© 2020 Brastianos HC et al. *JAMA Network Open*.

Continued:	**Full Model**		**Stepwise Model**[1]	
	HR (95% CI)	P-value	HR (95% CI)	P-value
Time between first melanoma diagnosis and first palliative treatment				
0-5 months	Reference		Reference	
6-11 months	1.26 (0.95-1.67)	0.103	1.27 (0.96-1.69)	0.092
12-17 months	1.00 (0.75-1.33)	0.976	1.01 (0.76-1.34)	0.958
18-23 months	0.83 (0.61-1.12)	0.216	0.83 (0.61-1.13)	0.231
24-35 months	0.99 (0.77-1.27)	0.951	1.00 (0.79-1.28)	0.974
36-59 months	1.09 (0.84-1.43)	0.518	1.09 (0.84-1.43)	0.509
60-119 months	0.78 (0.59-1.02)	0.070	0.78 (0.60-1.02)	0.067
120+ months	0.85 (0.64-1.12)	0.248	0.83 (0.62-1.10)	0.194
Time between first palliative treatment and first brain-directed treatment				
At first palliative treatment	Reference		Reference	
0-2 months	1.58 (1.19-2.09)	0.001	1.58 (1.19-2.09)	0.001
3-5 months	1.53 (1.16-2.03)	0.003	1.55 (1.17-2.05)	0.002
6-11 months	1.32 (1.02-1.73)	0.039	1.35 (1.03-1.76)	0.027
12+ months	1.09 (0.87-1.38)	0.448	1.10 (0.87-1.38)	0.423

Notes:
1. In the stepwise model, a significance level of 0.20 was used for model selection and variables with a significance level of 0.10 remained in the final model
HR = hazard ratio, CI = confidence interval

© 2020 Brastianos HC et al. *JAMA Network Open*.

eFigure 1. Timeline for Changes in Evidence and Recommendations Relevant to Melanoma Brain Metastasis Systemic Therapy and Radiotherapy

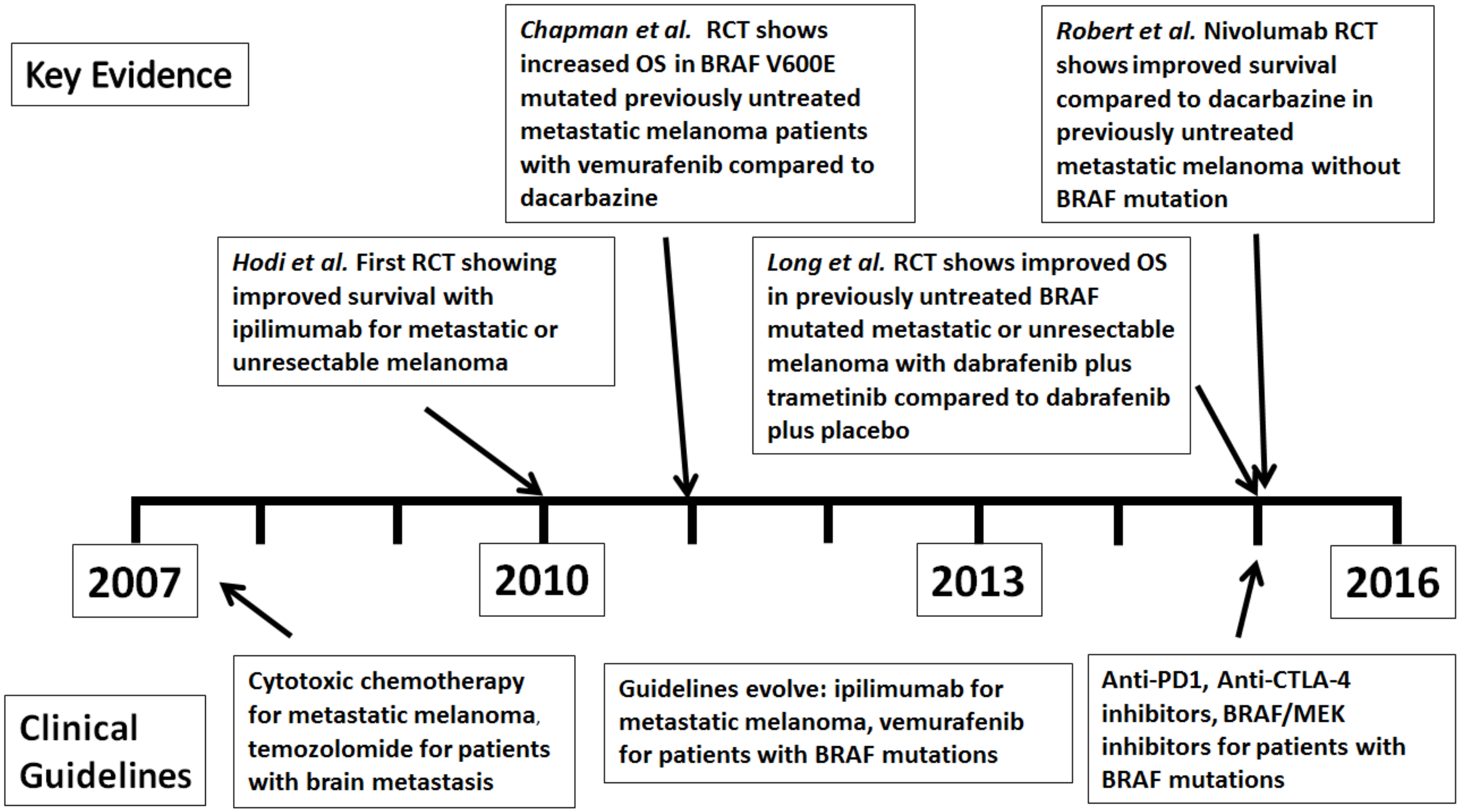

Supplementary eFigure 1a: Timeline for changes in evidence and guidelines for systemic therapy of metastatic melanoma and melanoma brain metastasis January 2007- December 2015. **OS** overall survival. Note that beyond this timeline, data on ipilimumab and nivolumab for melanoma brain metastases was first published in 2018 (Long et al, Tawbi et al).

© 2020 Brastianos HC et al. *JAMA Network Open*.

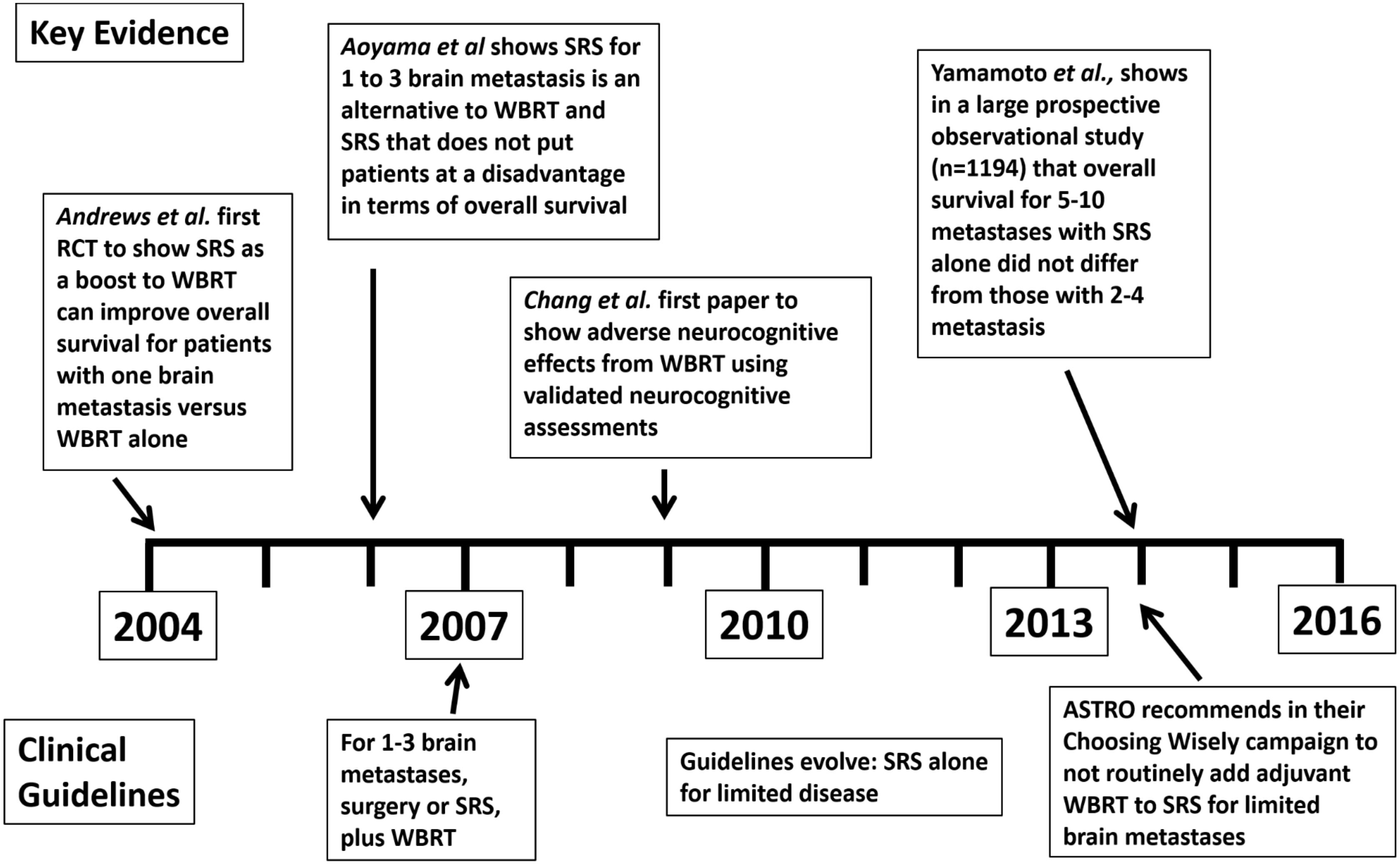

Supplementary eFigure1b: Timeline for changes in evidence and guidelines for radiotherapy of melanoma brain metastasis January 2004-December 2015. Note that beyond this timeline, in 2019, Hong et al showed that after local treatment of one to three melanoma brain metastases, adjuvant WBRT does not provide clinical benefit in terms of distant intracranial control, survival, or preservation of performance status. **WBRT** whole brain radiation therapy **SRS** stereotactic radiosurgery.

© 2020 Brastianos HC et al. *JAMA Network Open*.

eFigure 2. Identification of Melanoma Patients Treated Locally for Brain Metastases With Radiotherapy or Surgery in Ontario From January 1, 2007, to June 30, 2016

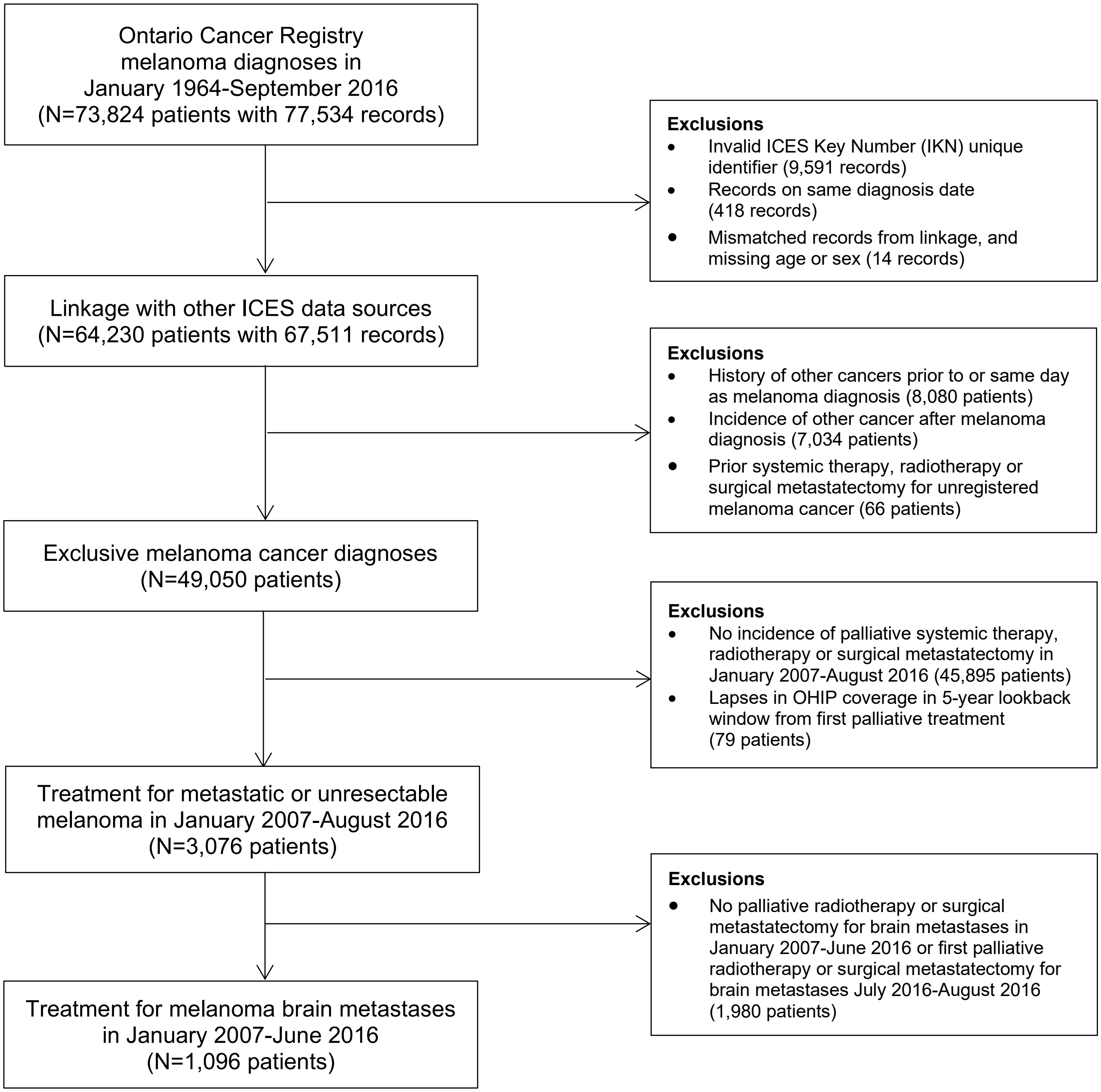

© 2020 Brastianos HC et al. *JAMA Network Open*.

eFigure 3. Overall Survival According to Period, Stratified by the Tertiles of Mean Number of Brain MRI Assessments per Patient by LHIN Health Region in the 3 to 9 Months Before the First Brain Treatment

(A) 1st tertile (lowest use); (B) 2nd tertile; (C) 3rd tertile (highest use). Survival curves not displayed beyond 1.25 years due to privacy regulations pertaining to groups of patients of five or less.

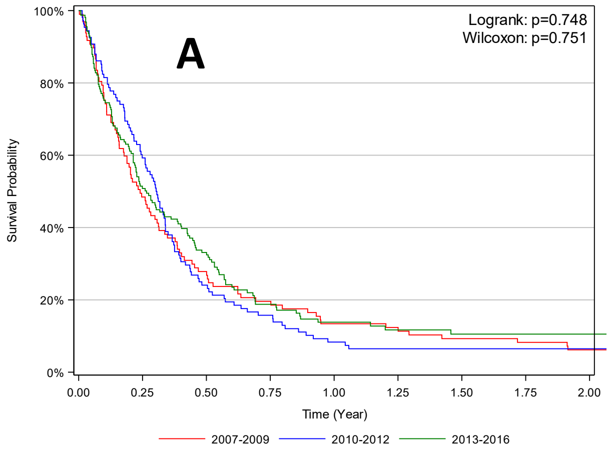

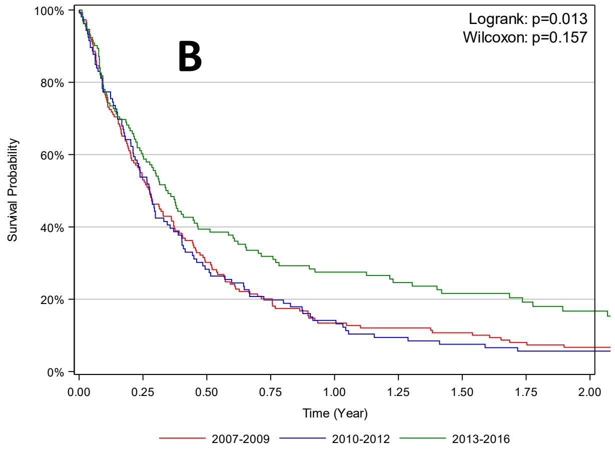

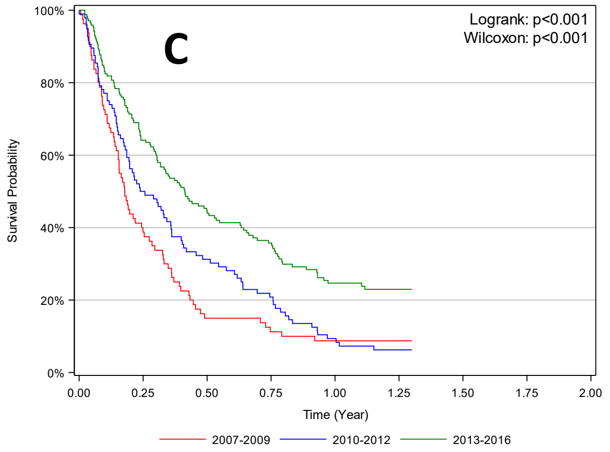

© 2020 Brastianos HC et al. *JAMA Network Open*.
